# Gartland type III extension supracondylar humerus fracture in a 10-year-old child. A surgical case report of an infrequent technique of medial and lateral column stabilization

**DOI:** 10.1016/j.ijscr.2023.109078

**Published:** 2023-11-19

**Authors:** S. Kiepura, J. Dutka

**Affiliations:** a Pediatric Surgery Department. Specialistic Hospital of Zeromski in Krakow, os. Na Skarpie 66, 31-913 Krakow, Poland; b Orthopedic Surgery Department. Specialistic Hospital of Zeromski in Krakow, os. Na Skarpie 66, 31-913 Krakow, Poland

**Keywords:** Supracondylar fracture, Children, Percutaneous fixation, Reduction

## Abstract

**Introduction and importance:**

Supracondylar humeral fractures in children are the most common fractures of the elbow accounting for 16 % of all pediatric fractures. The treatment depends on age, the degree of displacement, and the presence of additional injuries.

**Presentation of case:**

A case reports a 10-year-old girl with a Gartland type III supracondylar humeral fracture accompanied by anterior interosseous nerve neurapraxia preoperatively. The patient was treated operatively with medial and lateral column cross-pinning using four K-wires due to unsatisfactory closed reduction and lateral pinning only. Follow-up examinations performed in 1 and 6 months postoperatively revealed a 10° flexion contracture of the elbow with good functional and radiological results otherwise.

**Clinical discussion:**

The main intervention was not focused on the AIN neuropraxia itself but on unsatisfactory closed reduction followed by cross-fixation with lateral pinning only. A standard anterior approach to visualize the fracture line, free interposing tissues, and perform stabilization was utilized. The unusual use of an additional medial pin formed a cross-frame to adequately support the medial cortex.

**Conclusion:**

Closed reduction and percutaneous pinning are the preferred treatment options for most displaced supracondylar fractures. The open reduction via anterior approach and pinning for Gartland type III fracture gives good outcomes. Medial pinning is mandatory in particular fracture patterns and in case of unsatisfactory closed reduction. In the presented case medial and lateral column cross-pinning technique using four K-wires guaranteed no subsequent displacement on follow-up assessment and good results.

## Highlights

• Supracondylar humerus fractures are the most common fractures in children.
• Closed reduction and pinning is the preferred treatment method
• Medial pinning is necessary for particular fracture patterns.

## 1 Introduction

Supracondylar humerus fractures in children are the most common fractures around the elbow accounting for 16 % of all pediatric fractures [1]. The fractures occur after a fall onto the outstretched hand with the elbow in full extension, thus in 97–99 % of cases in extension type injury. The urgent neurovascular assessment should be done. Closed reduction and percutaneous pinning remain the most widely recognized treatment options for displaced supracondylar fractures. The open reduction is considered preferable in comminution, open fractures, or in case of neurovascular compromise [2,3]. Open reduction can be performed through the posterior, lateral, medial, or anterior approach, or a combination of these. Poor visibility through medial and lateral approaches and bad postoperative range of motion in the cases exposed posteriorly were reported [4,5]. The ideal approach should be safe, easy to perform with the ability to perform satisfactory reduction. In our center, the anterior approach, which was first described by Hagenbeck in 1894 is routinely performed to address pediatric supracondylar fractures. This approach provides exposure of the fracture line which facilitates protection liable to traumatic lesion structures. [6,7]. This paper presents a case report of a 10-year-old girl hospitalized with Garland type III completely displaced supracondylar humeral fractures accompanied by anterior interosseous nerve neurapraxia preoperatively. The child was treated operatively with medial and lateral column cross-pinning using four K-wires although the use of four K-wires is unusual as the vast majority of supracondylar fracture is stabilized by two or three K-wires. The main intervention was not focused on the AIN neuropraxia itself but on unsatisfactory closed reduction followed by cross-fixation with lateral pinning only.

## 2 Presentation of case

**Fig. 1:**
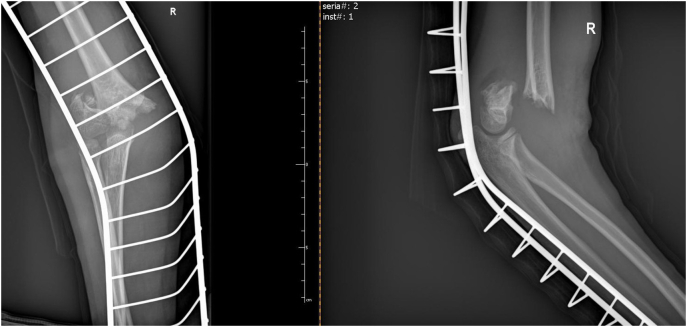
Preoperative radiograph of supracondylar humerus fracture. AP and lateral view.

**Fig. 2:**
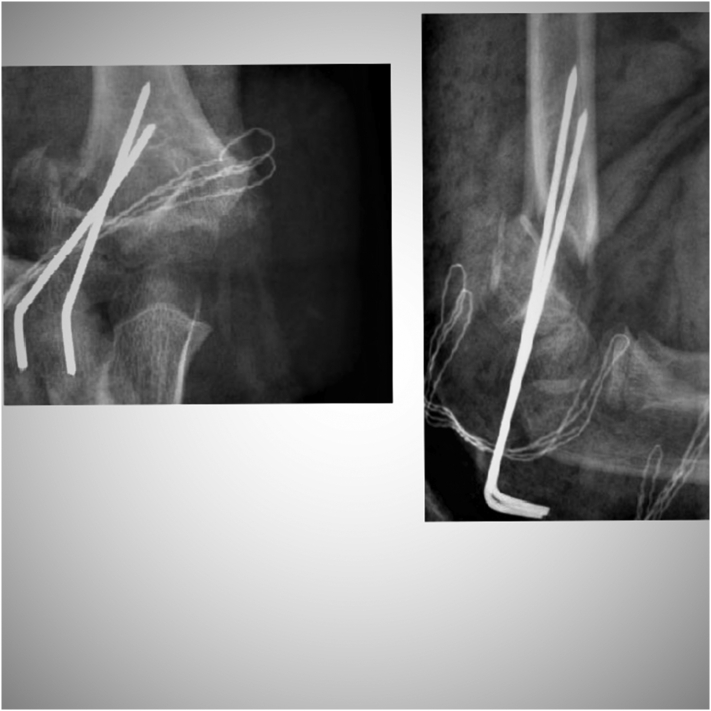
Intraoperative radiograph. Unsuccessful closed reduction and lateral pinning. AP and lateral view.

**Fig. 3:**
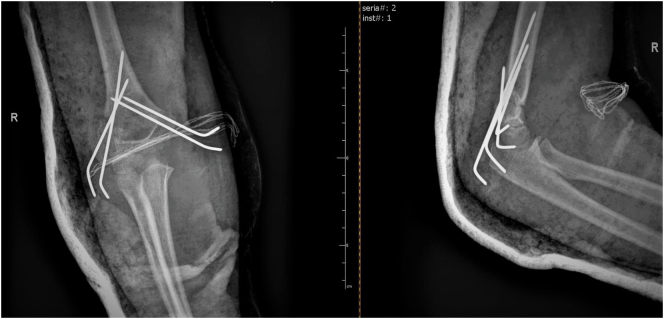
Postoperative radiograph. Open reduction with lateral and medial column pinning. AP and lateral view.

**Fig. 4:**
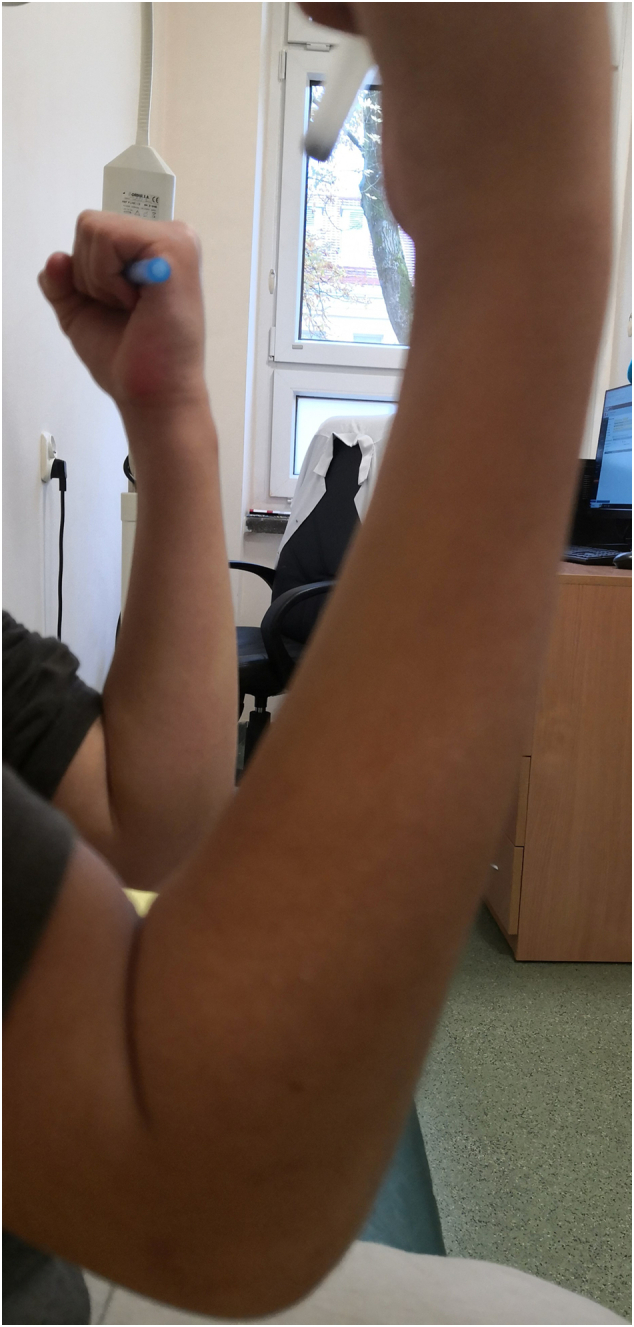
Post-op physical examination. Loss of 10° of elbow flexion. Lateral view.

**Fig. 5:**
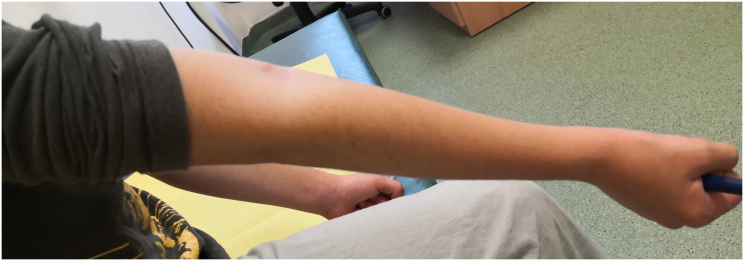
Post-op physical examination. Full extension of the affected elbow. Lateral view.

A 10-year-old girl was admitted to the Pediatric Surgery Department after falling onto an outstretched hand. The radiographs (in two planes including the wrist and elbow joint) revealed a completely displaced supracondylar humerał fracture - Gartland type III (Fig. 1). On physical examination, extensive edema and hematoma of the elbow joint with deformation and limitation of range of motion of the forearm were appreciated. The affected upper extremity presented the symptoms of anterior interosseous nerve (AIN) neurapraxia. The patient was otherwise well with no comorbidities and urgent surgical treatment was scheduled. The procedure was performed by a specialist pediatric orthopedic surgeon under general anesthesia and image intensifier. The patient was positioned supine with the affected arm placed on a radiolucent arm table. The image intensifier was positioned perpendicular to the arm, The affected extremity was prepared and draped in a standard manner and an Esmarch tourniquet was inflated. Initially, closed reduction was attempted by applying longitudinal traction to the forearm in line with the humerus in slight flexion, avoiding full extension to prevent tethering of neurovascular structures. After a graduał traction, a milking maneuver was performed to release the proximal fragment. Then hyperflexion of the elbow while pushing the olecranon in the anterior direction with the forearm in pronation to address internal rotation was performed. The fracture was initially stabilized percutaneously by 2 laterally placed K-wires. The reduction was assessed fluoroscopically using transcondylar views to assess the alignment of the medial and lateral columns of the elbow. The loss of reduction with a wide gap of fracture site on the medial view was seen so the decision of open reduction was made (Fig. 2). An anterior approach to the elbow was chosen. A median nerve and cubital vessel were dissected and protected on vessel loops. The proximal fragment was freed and reduced under direct vision. A standard technique of percutaneous pinning was performed with two 1.6 mm K-wires placed through the capitellum parallel to the fracture site and engaging the far cortex. Due to medial instability, an additional two K-wires were introduced from the medial epicondyle with protection on the ulnar nerve and engaged the opposite far cortex to form a cross-pinning construct. After pinning a sagittal alignment and stability of reduction and fixation were acceptable so the pins were carefully bent, cut, and left above the skin. The wound was closed in layers and the dressing was applied (Fig. 3). The above-elbow cast was shortened to the below elbow after 3 weeks to facilitate elbow rehabilitation. A kinesitherapy and iontophoresis course was commenced. Radiological control was obtained 1 and 3 weeks post-op and at the time of the final medical assessment. The K-wires were removed on an outpatient clinic basis after 4 weeks. The work has been reported per the SCARE criteria [8]. During the follow-up examination, a clinical and radiological assessment was made based on the Mayo Elbow Performance Score: pain, range of motion, joint stability, and functionality. Scores of 90–100 points were assessed as very good, 75–90 - good, 60–74 - average, and below 60 - bad [9]. The assessment was aimed at evaluating the correct alignment of the bone fragments, the presence of a bone union, cross-union between radius and ulna, or the formation of exostosis. The elbow joint was assessed for pain, range of motion, stability, and functionality. A score of 78 points was obtained according to the Mayo Elbow Performance Score, which gave a good treatment result. Radiological assessment after 4 weeks showed features of bone union, with the correct position of the bone fragments and no residual angular deformation. The child returned to normal physical activity 10 weeks postoperatively. A follow-up at 6 months after the fracture showed a 10° flexion contracture of the elbow with otherwise painless movement and full function (Fig. 4, Fig. 5). Neurovascular deficit or deep infection was not reported.

## 3 Discussion

Supracondylar humeral fractures in children can lead to impairment of the function of the elbow joint [10]. Conservative treatment with cast immobilization is suggested for minimally displaced or non-displaced fractures [10]. Closed reduction with or without pinning should be the treatment of choice in most cases nevertheless it depends on the experience of the surgeon and the characteristics of the fracture [[11], [12], [13]]. In children, open exploration is reserved for cases of closed reduction failure, multi-fragmentary fractures, neurovascular impairment, and coexisting fractures in the area of the elbow joint. A standard anterior approach to the elbow is generally thought as the most appropriate to visualize the fracture line, free interposing tissues, and preformed stabilization. The anterior approach provides direct access to interposed soft tissue in irreducible extension-type fractures. The median nerve and brachial artery can be identified and protected with vessel loops. In fractures with posteromedial displacement, the radial nerve is identified between the brachialis and brachioradialis at the lateral aspect of the wound and protected before attempting fracture reduction. In our case, the main intervention was not focused on the AIN neuropraxia itself but on unsatisfactory closed reduction followed by fixation with lateral pinning only as neuropraxia is commonly approached conservatively. In a study by Khademolhosseini et al. nerve injury rate is reported up to 18 % (only 3 % preoperative) which highlights the importance of a thorough preoperative and postoperative clinical examination of the patient [14]. Nevertheless, all patients sustained a nerve impairment in that series fully recovered at long-term follow-up. In the stabilization technique, an all-lateral fixation construct or cross-pinning fixation construct can be utilized. Huahuo et al. in their paper concluded that the crossed pinning reduced the loss of reduction risk [15]. Kocher et al. in the randomized prospective study reported a significant decrease in the risk of loss of reduction using the cross-pinning technique (4 %) compared with the all-lateral technique (21 %). In case of medial instability, an additional medial pin to support the medial cortex is required [16]. In the presented case all-lateral fixation was insufficient due to medial instability and inadequate cross-sectional area resulting from a steep fracture line. Edmonds et al. reported two more fracture pattern that necessitates a medial pin for the stability of the fixation: traverse fracture near the olecranon fossa and fractures with initial cubitus varus. In our case, the use of two parallel K-wires medially instead of one resulted in better fracture stabilization in the presented steep configuration of the fracture line. After inserting the first K-wire, satisfactory bone fragment compression and stability were not achieved. The operating surgeon introduced an additional wire from the medial side under visual control and an image intensifier. The additional wires would not harm the healing process nor damage the growth plate, and would not deteriorate the treatment process. Recovery of motor nerve injury is also a significant concern for authors. In a paper presented by Shore et al., the majority of nerve injuries associated with pediatric supracondylar humerał fractures recover within 6 months without acute nerve decompression [17]. It can be concluded that the open reduction via anterior approach and pinning are safe and gives good result in long-term observation, without leading to significant impairment of function or deformation of the elbow joint which corresponds to the data in the literature.

## 4 Conclusions

Closed reduction and percutaneous pinning are the preferred treatment options for most displaced supracondylar fractures. The open reduction via anterior approach and pinning for Gartland type III fracture gives good outcomes without leading to impairment of function or deformation of the elbow joint. Medial pinning is mandatory in particular fracture patterns and in case of unsatisfactory closed reduction. In the presented case medial and lateral column cross-pinning technique using four K-wires guaranteed no subsequent displacement on follow-up assessment and good results.

## Ethical approval

The patients' parents were informed that data from the research would be submitted for publication and gave their consent.

## Funding

None declared.

## CRediT authorship contribution statement

Study Design A

Data Collection B

Statistical Analysis C

Data Interpretation D

Manuscript Preparation E

Literature Search F

Funds Collection G

1. Author: Slawomir Kiepura, M.D, ABCDEF

2. Co-author: Julian Dutka, Ph.D., ADE

## Guarantor

Julian Dutka.

## Parental consent

Written informed consent was obtained from the patient's parents/legal guardian for publication and any accompanying images. A copy of the written consent is available for review by the Editor-in-Chief of this journal on request.

## Declaration of competing interest

None declared. The authors have no financial, consultative, institutional, and other relationships that might lead to bias or conflict of interest.
